# Dynamics of DNA Origami Lattices

**DOI:** 10.1021/acs.bioconjchem.2c00359

**Published:** 2022-09-15

**Authors:** Sofia Julin, Adrian Keller, Veikko Linko

**Affiliations:** † Biohybrid Materials, Department of Bioproducts and Biosystems, Aalto University, 00076 Aalto, Finland; ‡ Paderborn University, Technical and Macromolecular Chemistry, Warburger Str. 100, 33098 Paderborn, Germany; § LIBER Center of Excellence, Aalto University, 00076 Aalto, Finland

## Abstract

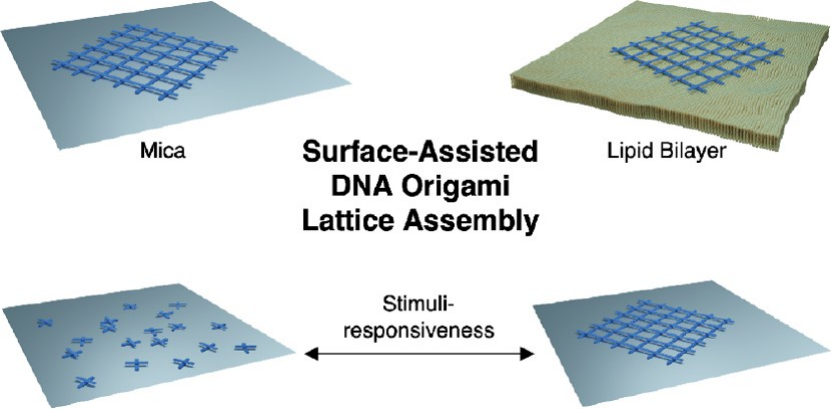

Hierarchical assembly of programmable DNA frameworks—such as DNA origami—paves the way for versatile nanometer-precise parallel nanopatterning up to macroscopic scales. As of now, the rapid evolution of the DNA nanostructure design techniques and the accessibility of these methods provide a feasible platform for building highly ordered DNA-based assemblies for various purposes. So far, a plethora of different building blocks based on DNA tiles and DNA origami have been introduced, but the dynamics of the large-scale lattice assembly of such modules is still poorly understood. Here, we focus on the dynamics of two-dimensional surface-assisted DNA origami lattice assembly at mica and lipid substrates and the techniques for prospective three-dimensional assemblies, and finally, we summarize the potential applications of such systems.

## 1 Introduction

DNA nanotechnology enables construction of accurate artificial structures from DNA molecules with arbitrary geometries and a high level of addressability.^1−3^ Therefore, these precise structures may serve as components in applications ranging from nanoelectronics^4^ to nanophotonics^5^ and from nanomedicine^6^ to inorganic materials engineering.^7^ The most widely used technique to assemble DNA nanostructures is DNA origami, which is based on folding a long single-stranded DNA (ssDNA) scaffold strand into a predefined shape with the help of dozens of short ssDNA molecules, unique in sequence.^8,9^ DNA origami is a robust method to create modular DNA motifs for versatile nanopatterning purposes^10,11^ as well as for higher-order self-assembly systems.^12,13^ Already before the invention of DNA origami, principles of hierarchical self-assembly of various other programmable DNA-based modules were introduced. For example, Seeman and co-workers introduced two-dimensional (2D) lattices using, e.g., Holliday junctions,^14^ double-crossover (DX)-tiles,^15^ and three-space-spanning DNA motifs^16^ as building blocks. Other lattice types have also been reported; so-called 4 × 4 tiles may assemble into nanoribbons and nanogrids^17^ and cross-shaped motifs into square-like arrays,^18^ while T-^19^ and Y-shaped DNA junctions^20^ may form continuous well-defined patterns through surface-assisted assembly.

In general, DNA-based hierarchical assembly is based on addressable, directional and fully programmable modification sites, which means that the “*valency”* of such motifs unambiguously guides the formation of higher-order structures.^21,22^ As indicated above, DNA origami has provided much more flexibility and freedom for designing precise DNA architectures. This absolute control of the valency makes DNA origami an optimal candidate for algorithmic assemblies and large-scale lattices. The dimensions of a conventional, single DNA origami object are limited to a range from a few nanometers to a few hundred nanometers (which is governed by the length of the scaffold strand), but along with the ever-advancing DNA origami design techniques,^23,24^ the increasing complexity of the structures,^25^ and the concurrent software development,^26−28^ a plethora of different strategies employing DNA origami in macroscopic assemblies have emerged. The various approaches to arrange lattices using different DNA motifs have been recently covered in multiple reviews.^12,13,29^

Here, instead of simply summarizing the achieved lattice types using DNA origami structures as building blocks, we focus on the dynamics of lattice assembly and reconfiguration. This covers surface-assisted high-order and long-range 2D DNA origami assemblies on both mica- and lipid-based substrates, dynamic assembly of three-dimensional (3D) origami lattices as well as stimuli-responsive assemblies and the potential applications of such systems. Note that in this review, we deliberately focus on 2D and 3D lattices. One-dimensional (1D) assemblies will only be mentioned if they allow for fundamentally different insights or can easily be scaled up to 2D or 3D.

## 2 Methods for Assembling DNA Origami Lattices

There are two conceptually different strategies for assembling individual DNA origami nanostructures into ordered lattices. In the first and most established one, lattice assembly is achieved in bulk solution. Therefore, this strategy relies on attractive interactions between the individual DNA origami, which additionally must be controlled in such a way that the assembly of ordered 2D or 3D lattices is favored over the formation of disordered, amorphous aggregates. For this, the earliest examples of DNA origami lattices reported by Rothemund^8^ and Liu et al.^30^ took advantage of sticky-end cohesion. Here, certain staple strands along the edges of the DNA origami were extended, and these “sticky ends” further hybridized to complementary sticky-end sequences protruding from other DNA origami. Due to the strong sequence-specificity of DNA hybridization, this approach also allows for the assembly of lattices with complex symmetries,^31^ defined boundaries and dimensions,^8^ or hybrid lattices composed of different DNA origami tiles.^32^

As an alternative to sticky-end hybridization, also blunt-end stacking facilitates the specific binding between individual DNA origami nanostructures.^33^ Blunt-end stacking occurs between truncated duplexes ending in solution-exposed base pairs (bp) and is well-known as a critical factor that may induce nonspecific DNA origami aggregation.^8^ Employing the concept of shape complementarity, however, enables the rational design of the intermolecular interactions, with regard to both interaction strength and specificity.^34^ Consequently, large and complex DNA origami assemblies^35^ and ordered lattices and crystals^33^ can be produced this way.

In addition, more exotic interactions can be exploited for the specific binding and arrangement of DNA origami nanostructures. These include for instance hydrophobic interactions,^36^ electrostatic interactions,^37,38^ host/guest interactions,^39^ and combinations thereof.^40^ However, these implementations typically require the introduction of non-DNA functional entities, either in the form of coassembling species^38^ or by covalent conjugation to selected staple strands.^36,39,40^

The second method is based on the assembly of 2D lattices using DNA origami monomers adsorbed at a solid–liquid,^41,42^ lipid–liquid,^43^ or liquid–air interface.^44^ The interface plays two roles in this strategy. First, attractive interactions between the DNA origami and the interface result in DNA origami adsorption and thereby reduces their degrees of freedom as their motions are now confined to a 2D plane. At the same time, however, it is important that the attractive interactions are weak enough to provide the adsorbed DNA origami monomers with sufficient lateral mobility to diffuse along the interface and assemble into an ordered lattes. Second, since DNA origami adsorption is energetically favored, a closed DNA origami monolayer will form over time when the interface is exposed to a sufficiently high DNA origami concentration. Maximum coverage of the interface will be obtained for a densely packed monolayer, which for a single DNA origami species with high symmetry such as a rectangle or a triangle is equivalent with an ordered lattice. Therefore, lattice assembly at interfaces may occur even in the absence of any attractive intermolecular interactions.^42^ However, introduction of such attractive interactions between individual DNA origami may result in more stable lattices, higher lattice order, or more complex arrangements.^41−43^

## 3 Dynamics of Surface-Assisted 2D Lattice Assembly at Mica Substrates

**Figure 1:**
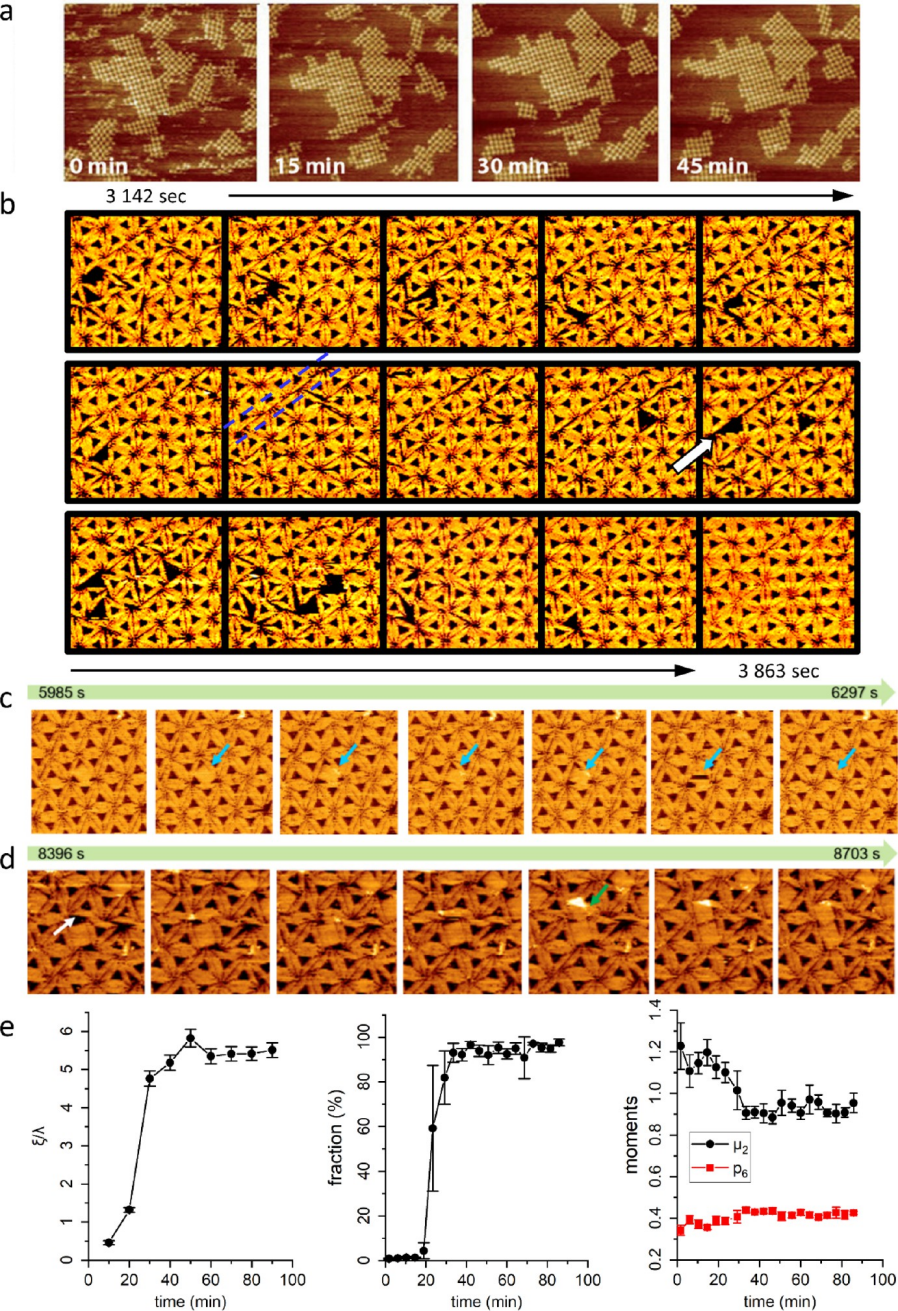
Dynamics of 2D DNA origami lattice assembly at mica surfaces. (a) Consecutive AFM images of a growing lattice of cross-shaped DNA origami nanostructures during assembly via blunt-end stacking (scan size 3 × 3 μm^2^). (b) Consecutive HS-AFM images (interval between images 51.5 s) showing the formation and annealing of a line defect (indicated by the blue broken lines) within a hexagonally ordered lattice of DNA origami triangles without any blunt-end stacking. (c,d) Consecutive HS-AFM images (interval between images 51.2 s) of the stimulated desorption (c) and dimer formation (d) of DNA origami rectangles (arrows) incorporated in lattices of DNA origami triangles at T:R ratios of 100:1 (c) and 10:1 (d), respectively. (e) Evolution of the relative correlation length ξ/λ, the fraction of DNA origami triangles in the largest connected cluster, and the topological parameters μ_2_ and *p*_6_ during the Ca^2+^-mediated assembly of a lattice of DNA origami triangles. All parameters were calculated from HS-AFM images. (a) Reprinted with permission from ref (42). Copyright 2014 John Wiley & Sons. (b) Reprinted with permission from ref (45). Copyright 2018 American Chemical Society. (c,d) Reprinted with permission from ref (48). Copyright 2020 Royal Society of Chemistry. (e) Reprinted with permission from ref (49). Published 2020 by Springer Nature.

The first 2D DNA origami lattices assembled at solid–liquid interfaces were presented in 2014 by the laboratories of Rothemund^41^ and Simmel.^42^ Both works utilized hierarchical DNA origami assembly at mica surfaces, building on earlier studies that employed smaller DNA tile motifs.^19,20^ The general approach is based on controlling the electrostatic interactions of the adsorbed DNA origami nanostructures and the mica surface. Since both the DNA origami and the surface are negatively charged, adsorption is facilitated by an intermediate layer of divalent Mg^2+^ ions. Upon addition of an excess of monovalent Na^+^ ions, some Mg^2+^ ions are displaced from the mica–DNA interface, resulting in reduced electrostatic attraction and thereby enhanced DNA origami mobility at the mica surface. This enables the DNA origami to diffuse along the surface and optimize their arrangement to accommodate more incoming DNA origami nanostructures and assemble into ordered 2D lattices. In this way, Aghebat Rafat et al. demonstrated ordered lattices assembled from DNA origami rectangles, triangles, and cross shapes with tetragonal, hexagonal, and herringbone symmetries, respectively.^42^ For the cross shapes, different lattices were obtained depending on whether their edges displayed blunt ends or not, the former resulting in attractive interactions between individual DNA origami monomers via blunt-end stacking. This led to the assembly of well-ordered and almost defect-free 2D DNA origami crystals a few μm in size. As can be seen in Figure 1a, lattice assembly was sufficiently slow to be followed in real-time using conventional atomic force microscopy (AFM). By employing DNA origami crosses with biotinylated staple strands, the assembled lattices could be further functionalized to display streptavidin. Woo and Rothemund used a similar approach to assemble more complex checkerboard lattices from DNA origami rectangles that featured blunt ends only at selected corner positions along their edges.^41^

Using high-speed AFM (HS-AFM), Kielar et al. investigated the dynamics of DNA origami lattice assembly at mica surfaces using DNA origami triangles without any blunt ends.^45^ In this case, lattice assembly occurs simply in order to accommodate the electrostatic requirements of the mica surface and minimize the exposed mica surface area. The result is a polycrystalline lattice with hexagonal symmetry that can homogeneously cover macroscopic surface areas of ∼10 cm^2^ and beyond.^46^ In their investigations, Kielar et al. focused on the effect of the Na^+^ concentration on the development of lattice order, which was quantified by calculating the correlation length ξ, i.e., the length within which the surface heights of any two points are correlated, from the 2D power spectral density of the recorded HS-AFM images.^47^ They found that maximum order is obtained at 75 mM Na^+^ in combination with 10 mM Mg^2+^. At lower Na^+^ concentrations, the mobility of the DNA origami triangles was too low to dynamically anneal lattice defects, whereas higher Na^+^ concentrations resulted in rapid surface diffusion and desorption of the triangles. The authors then investigated the formation and annealing of lattice defects at the optimum Na^+^ concentration of 75 mM. They observed that zero-dimensional (0D) point defects composed of damaged DNA origami triangles can be annealed rather easily if the damaged triangle has a smaller contact area with the mica surface than the intact ones. In this case, an incoming intact triangle can replace the damaged one in the lattice. Annealing of larger 1D line defects is also possible but requires the concerted rearrangement of many DNA origami nanostructures in the vicinity (see Figure 1b). 2D screw-like dislocations turned out to be the most stable defects that form at rather late stages of assembly and are almost impossible to anneal. These types of complex defects thus limit the overall order that can be achieved.

In a follow-up study, Xin et al. deliberately introduced defects into the hexagonally ordered DNA origami lattice by adding a small fraction of DNA origami rectangles to the triangles.^48^ At high to moderate triangle-to-rectangle (T:R) ratios of 100:1 or higher, rectangles were incorporated at low densities into the assembling hexagonal lattice but later on efficiently replaced by other triangles, because the lateral strain of the surrounding lattice stimulated their desorption and their nonfitting shape provided incoming triangles an opportunity to make contact with the mica surface (see Figure 1c). Most astonishingly, at such a moderate T:R ratio of 100:1, the resulting lattice had a larger correlation length than at a higher T:R ratio of 200:1. This was rationalized by the nonmatching shape of the rectangles blocking larger surface areas than the triangles, which leads to increased mobility of neighboring triangles and thereby the more efficient annealing of other defects. At lower T:R ratios of 10:1 and lower, the incorporated rectangles persisted longer within the lattice and even started to form dimers and multimers (see Figure 1d). Even under those conditions, however, the lattices proved resilient with regard to the incorporation of rectangles.

In order to further improve the achievable degree of lattice order, Xin et al. screened the effect of different monovalent and divalent cation species on the assembly of DNA origami triangles into hexagonal lattices.^49^ They observed that due to their different ways of binding to DNA and mica, Li^+^ and K^+^ were inferior to Na^+^ in facilitating the assembly of highly ordered lattices. Substituting Mg^2+^ for Ca^2+^, however, resulted in significantly enhanced lattice order, which was explained by the weaker binding of Ca^2+^ to the DNA phosphates, so that it can be displaced more easily by the Na^+^ ions. For this combination of cations, the authors also investigated the development of order during lattice assembly using a number of different order-related parameters, i.e., the relative correlation length ξ/λ, which has been normalized to the lattice periodicity λ, the fraction of DNA origami triangles in the largest connected cluster, and the topological parameters μ_2_ and *p*_6_, which indicate the variance of the distribution of nearest neighbors and the relative proportion of DNA origami triangles with exactly six neighbors, respectively.^50,51^ All of these parameters were calculated from the recorded HS-AFM images, using either the power spectral density function^47^ or the Delaunay triangulation^52^ of the lattices. All of these parameters indicated a rapid increase of order within the first 30 min of incubation, i.e., during the time it takes to form a closed DNA origami monolayer, whereas longer incubation times resulted in a much slower and more gradual increase in order (see Figure 1e). Finally, the authors demonstrated that combining all independently optimized assembly parameters, i.e., cation concentrations, cation species, DNA origami concentration, and assembly time, highly ordered lattices with unprecedented correlation lengths beyond 8λ can be obtained.

## 4 Dynamics of Surface-Assisted 2D Lattice Assembly at Lipid Bilayers

**Figure 2:**
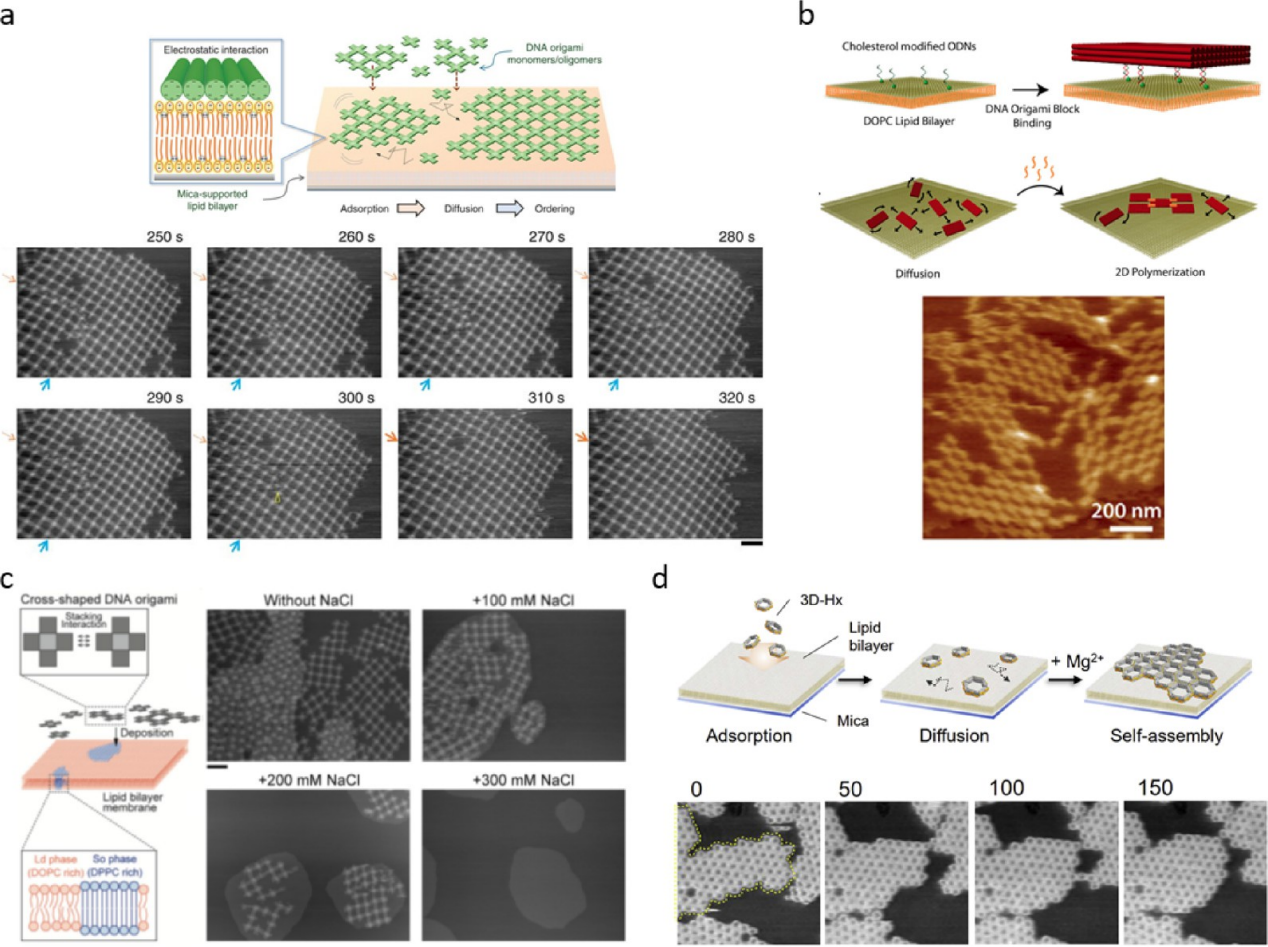
DNA origami lattice assembly at supported lipid bilayers (SLBs). (a) Lattice assembly by electrostatic adsorption of DNA origami onto a SLB. Consecutive HS-AFM images (scale bar 200 nm) demonstrating the lattice assembly and the fusion of two domains at the boundary region indicated by orange and blue arrows. (b) Top panel: Cholesterol molecules are used to anchor rectangular DNA origami units onto the lipid bilayer before the addition of the oligonucleotides that connect the units into a 2D lattice. Bottom panel: AFM image of the assembled lattice. (c) Right panel: 1,2-dioleoyl-*sn*-glycero-3-phosphocholine (DOPC) and 1,2-dipalmitoyl-*sn*-glycero-3-phophocholine (DPPC) is used to prepare a phase-separated SLB. Left panel: As shown in the AFM images (scale bar 200 nm), the cross-shaped DNA origami adsorbs differently on the fluidic liquid-disordered (Ld) and solid-ordered (So) phases. (d) Shape-complementary hexagonal DNA origami stack together and assemble into a 2D lattice when the Mg^2+^ concentration is increased. The lattice growth is followed by HS-AFM (image size 800 nm × 800 nm). (a) Reprinted with permission from ref (43). Published 2015 by Springer Nature. (b) Reprinted with permission from ref (60). Copyright 2015 American Chemical Society. (c) Reprinted with permission from ref (58). Copyright 2018 John Wiley & Sons. (d) Reprinted with permission from ref (59). Published 2022 by Cell Press.

Supported lipid bilayers (SLBs) are widely used to mimic biological membranes and to study membrane-associated processes and interactions.^53,54^ SLBs are prepared on mica or other substrates by the adsorption and self-assembly of lipid molecules, such as the synthetic zwitterionic lipid 1,2-dioleoyl-*sn*-glycero-3-phosphocholine (DOPC). This lipid is often used either alone or in combination with other lipids for SLB formation.^43^ The mica-SLBs are flat and dynamic surfaces for which the fluidity and surface charge readily can be altered by the lipid composition, and therefore, these are also suitable for surface-assisted self-assembly of DNA origami-based lattices.^55^ DNA origami can be adhered to the lipid bilayer surface purely through electrostatic interactions (see Figure 2a) or by functionalizing DNA origami with hydrophobic molecules that further interact with the lipids in the bilayer membrane (see Figure 2b).^56^ The latter strategy allows for a more selective attachment as the position of the hydrophobic moieties provides control over DNA origami orientation in the lipid bilayer. The electrostatic adsorption of the DNA origami onto the SLBs is, similar to mica, also mediated by divalent ions, such as Mg^2+^. In the case of the commonly employed zwitterionic DOPC bilayers, the interactions are weak enough so that the DNA origami structures retain their mobility on the substrate, and thus they may assemble into well-ordered 2D lattices. This was first demonstrated by Suzuki et al., who assembled micrometer-sized lattices using cross-shaped DNA origami structures that were electrostatically adsorbed on a DOPC bilayer in plain folding buffer (10 mM Mg^2+^) and connected at the edges through blunt-end interactions.^43^ Similarly, they also assembled closed-packed lattices using symmetric DNA origami structures without any blunt-ends, i.e., the same cross-shaped DNA origami structure, a DNA origami triangle, and a DNA origami hexagon. Remarkably, an addition of either of the monovalent ions, Na^+^ or K^+^, resulted in a detachment of the assembled lattice when some of the Mg^2+^ ions were replaced by the monovalent ions thus weakening the interaction between the DNA origami and the DOPC bilayer. Furthermore, by studying the self-assembly on the DOPC bilayer by HS-AFM, they observed that the process is dynamic—monomers and multimers at the boundary between adjacent lattices frequently associated and dissociated in order to assemble into larger uniform lattices (see Figure 2a). Interestingly, they also noticed that point defects in the lattice can be healed by adding free monomers to the system, similar to the case of mica surfaces. This was later utilized in another study by the same research group when they demonstrated that the cavities in a preassembled lattice composed of cross-shaped DNA origami can be filled up with square-shaped DNA origami structures.^57^ The incorporation of the square-shaped DNA origami into the cavity was found to be a highly reversible process with repeated adsorption and desorption of DNA origami, and the system could be further fine-tuned by adjusting the Mg^2+^ concentration. A high DNA origami population inside the cavities was observed only at remarkably high Mg^2+^ concentrations (50 mM Mg^2+^) or by introducing complementary DNA-strands that connected the square-shaped DNA origami to the preassembled lattice through hybridization.

The behavior and mobility of the DNA origami on the SLBs is highly dependent on the fluidity and surface charge of the lipid bilayer, which can be easily modified through the lipid composition.^58^ Sato et al. studied the DNA origami lattice assembly on a phase-separated lipid bilayer prepared as a mixture of unsaturated DOPC and saturated 1,2-dipalmitoyl-*sn*-glycero-3-phophocholine (DPPC) that forms fluidic liquid-disordered (Ld) and solid-ordered (So) phases, respectively. At high DNA origami concentrations, they observed that the cross-shaped DNA origami bound to the Ld-phase assembled into micrometer-sized lattices, whereas DNA origami on the So phase were prone to aggregation due to the higher surface charge and lower fluidity (see Figure 2c). Addition of Na^+^ ions detached the DNA origami completely from the Lo phase, while on the So phase the higher rate of DNA origami association and dissociation in combination with higher DNA origami surface mobility resulted in a reorganization of the aggregates into well-ordered lattices.

Conventionally, lattices on SLB and mica substrates have been assembled using 2D DNA origami structures, but recently it was demonstrated that also three-dimensional (3D) DNA origami structures can be utilized for lattice assembly on SLBs.^59^ In this work, hexagonal DNA origami blocks with shape-complementary interfaces were assembled into predefined 2D lattices on a DOPC bilayer. To assemble highly ordered monolayers, the DNA origami hexagons were adsorbed onto the SLB at a relatively low Mg^2+^ concentration (15 mM), after which the Mg^2+^ concentration was increased to 50 mM in order to reduce the electrostatic repulsion at the interfaces and thus allowing the DNA origami units to stack together. Notably, such a high Mg^2+^ concentration would not be suitable for lattice assembly on mica since it would make the DNA origami structures immobile on the surface. The authors also followed the lattice assembly with HS-AFM and observed that adsorption and desorption of the DNA origami units not only occurred at the edges of the lattice, but also at the defect sites inside the lattice, thus resulting in defect splitting, defect diffusion, and defect filling (see Figure 2d).

As already mentioned, the DNA origami can also be attached to the SLBs by utilizing hydrophobic moieties that anchor DNA structures onto the lipid membrane. By functionalizing the DNA origami with cholesterol molecules interacting with the DOPC bilayer, Kocabey et al. assembled a rectangular DNA origami block that could assemble either into 1D chains or 2D lattice patterns depending on which polymerization oligonucleotides were used (see Figure 2b).^60^ Similarly, they also used a Y-shaped DNA origami unit to assemble a hexagonal lattice. With this assembly strategy, the DNA origami objects have a predefined orientation in the lipid bilayer and are restricted to diffusion only in 2D, which allows the assembly of lattices that are an order of magnitude larger than similar lattices assembled in solution. Furthermore, the interaction between the DNA origami and the lipid bilayer—as well as the DNA origami diffusion on the lipid membrane—can be easily modulated by the size and number of the cholesterol molecules. In a later study, it was demonstrated that the DNA origami diffusion rate on SLBs can also be controlled by the ionic strength of the solution.^61^ Interestingly, it was observed that the DNA origami diffusion rate is dependent on whether the SLB is prepared on glass or on mica. On a glass-SLB, the DNA origami mobility was completely retained for most DNA origami structures in a buffer containing 5 mM Mg^2+^, whereas DNA origami on a mica-SLB were still mobile even at Mg^2+^ concentrations of 50 mM.

## 5 Stimuli-Induced Dynamics of DNA Origami Lattices

### 5.1 Dynamic Lattice Assembly and Disassembly

There are a number of different approaches for directing the assembly and disassembly of DNA origami-based lattices with external stimuli, such as pH, salt concentration, light, and temperature. For example, Zhang et al. used shape-complementary blunt ends at the interfaces to polymerize DNA origami “tensegrity triangles” into a 3D rhombohedral lattice at a constant temperature (47 °C).^33^ The base stacking interactions at the interfaces are temperature-dependent and by increasing the temperature to 50 °C, the interactions were already so weak that the lattice was completely disintegrated. Similarly, temperature could also be used to guide the assembly of DNA origami lattices formed through sticky-end cohesion. Lin et al. used a set of distinct sticky ends to connect DNA origami “nanochambers” into 1D, 2D, and 3D lattices in a programmable manner.^62^ The melting temperature of the hybridization strands correlated directly with their length, and the authors observed that a certain sticky-end length (8 bp) was required for the successful assembly of lattices. Shorter sticky ends (6 bp) resulted in unconnected monomers, whereas longer sticky ends (10 bp) yielded only large, kinetically trapped aggregates. As a different approach, Julin et al. took advantage of the ionic strength of the solution to control the assembly of negatively charged DNA origami 6-helix bundles and cationic gold nanoparticles (AuNPs) into highly ordered lattices.^38^ At high ionic strengths, the electrostatic interactions between the building blocks are screened and no assemblies are formed. However, when the salt concentration is gradually decreased, the compounds will coassemble into well-ordered 3D tetragonal superlattices.

**Figure 3:**
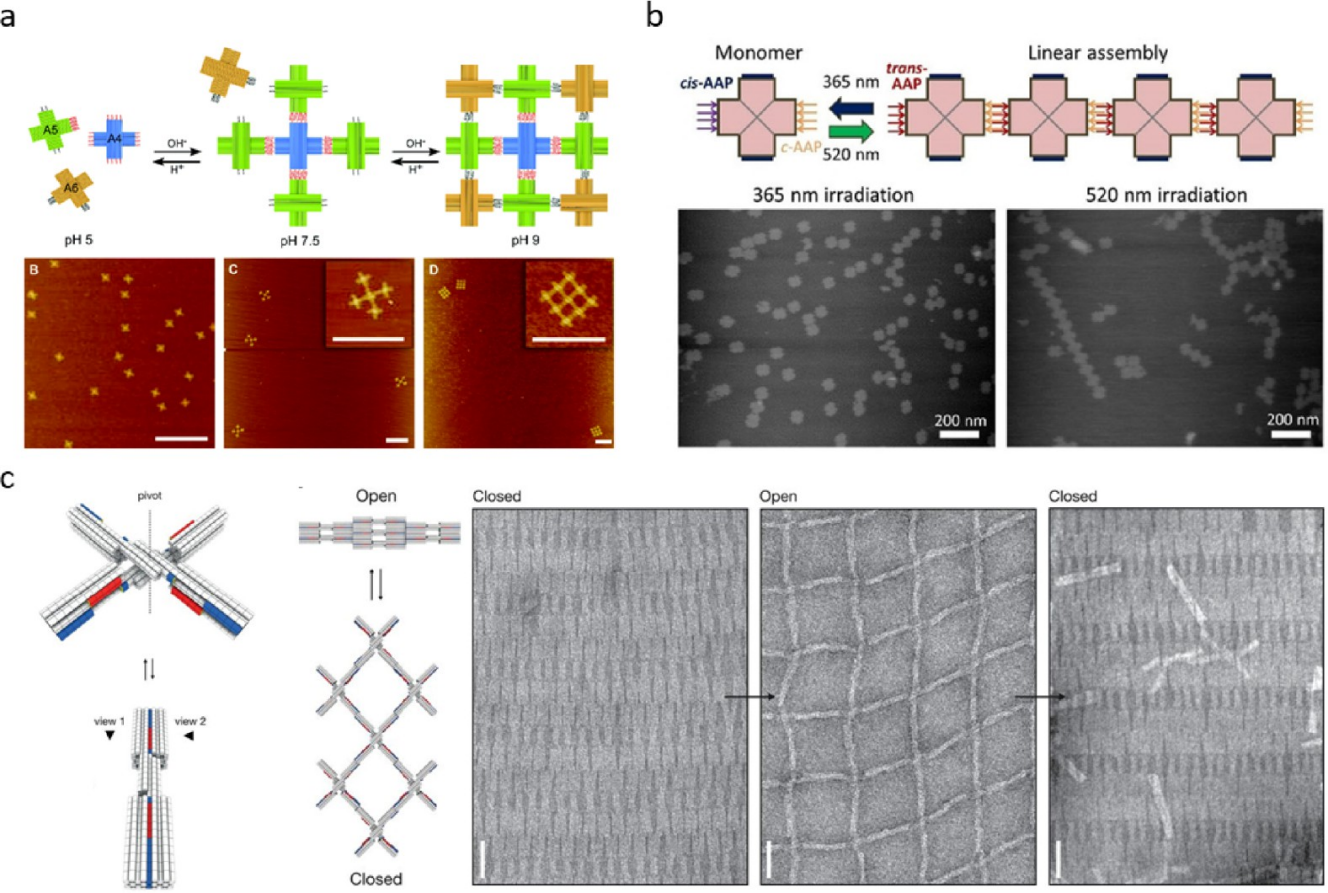
Stimuli-induced dynamics of DNA origami lattices. (a) The pH-responsive and stepwise assembly and disassembly of DNA origami nanoclusters is monitored by AFM (scale bars 500 nm). (b) The polymerization of X-shaped DNA origami into linear chains is controlled by photosensitive arylazopyrazole-modified oligonucleotides. The AFM images show the disassembled and assembled structures after irradiation at 365 and 520 nm, respectively. (c) Left panel: Reconfigurable DNA origami switch with shape-complementary protrusions and recessions allowing it to change conformation depending on the salt concentration. Right panel: The reconfigurable lattice constructed by connecting several switch units could readily and reversibly shrink and grow as demonstrated in the TEM images (scale bar 50 nm). (a) Reprinted with permission from ref (63). Copyright 2019 The Royal Society of Chemistry. (b) Reprinted with permission from ref (68). Copyright 2021 John Wiley & Sons. (c) Reprinted with permission from ref (34). Copyright 2015 The American Association for the Advancement of Science.

There also exist other stimuli-induced approaches for controlling the assembly and disassembly of DNA origami arrangements that could be potentially scaled up and, therefore, equally used for 2D and/or 3D lattices. For example, recently, pH-sensitive DNA triplexes were used to selectively and stepwise regulate the association of nanoclusters containing nine cross-shaped DNA origami units (see Figure 3a).^63^ By increasing the pH, the DNA triplex will dissociate, and the freed ssDNA domain of the triplex can further be used as one of the hybridization strands connecting the units together. In a similar manner, pH-responsive i-motif and triplex DNA sequences were used to control the assembly and disassembly of DNA origami dimers and trimers.^64^ In addition, photoresponsive molecules have been used to control the hybridization of adjacent DNA origami units and thereby the assembly and disassembly of a cornucopia of arrangements.^65−68^ Yang et al. used azobenzene-modified oligonucleotides to connect hexagonal DNA origami units together into 1D chains and ring-shaped hexamers and could reversibly direct the association and dissociation by irradiating the sample with visible light and ultraviolet light, respectively.^65^ In a subsequent study, another photoswitchable molecule, arylazopyrazole, was used for the photocontrolled assembly and disassembly of X-shaped DNA origami into linear assemblies (see Figure 3b).^68^ In addition to these, ssDNA fuel and antifuel strands^69^ and modular DNA strand displacement cascades^70^ have been used as external triggers for the assembly and disassembly of linear DNA origami fibrils.

### 5.2 Dynamic Lattice Reconfiguration

The focus of structural DNA nanotechnology is currently shifting from static arrangements to dynamic structures that can change their conformation in response to environmental stimuli. In general, the synthesis of dynamic nanomaterials is rather challenging, but a variety of dynamic miniature devices has been successfully built using the DNA origami technique.^71,72^ However, the use of DNA origami for the construction of larger dynamic assemblies is still rather limited, but a few selected and appealing approaches are discussed here in more detail.

Gerling et al. constructed a reconfigurable micrometer-sized 2D lattice utilizing cross-like DNA origami switches that were connected through hybridization (see Figure 3c).^34^ The switch has shape-complementary interfaces on the arms, and by taking advantage of the weak base stacking interactions between the predesigned protrusions and recessions, the switch could adopt an open or a closed configuration depending on the salt concentration of the surrounding solution. At high salt concentration, the electrostatic repulsion between the two arms of the switch is overcome, and the unit is locked into a closed state, but the arms are readily released again when the ionic strength is decreased. Thus, the lattice assembled from this DNA origami switch could be reversibly expanded and squeezed simply by changing the ionic strength of the solution.

DNA origami lattices connected with sticky ends are rather sensitive to environmental factors. For example, it has been demonstrated that high salt concentration, ethanol and certain polymers may cause a shrinkage of the ssDNA region and thereby a contraction of the whole DNA origami lattice.^73^ In a recent work, Wang et al. substituted the spacing region with a flexible and pH-sensitive i-motif sequence that could form a C-quadruplex structure at low pH.^74^ The C-quadruplex formation resulted in a shortening of the distance between adjacent DNA origami units, but the original interunit distance could be recovered by increasing the pH and thus turning the C-quadruplex back to ssDNA counterparts. The i-motif was incorporated into the connector sequences used to link DNA origami units into a 3D lattice, and depending on whether the i-motif was part of the connector strand in 1D, 2D, or 3D, the lattice could rapidly and reversibly either switch between a simple cubic and simple tetragonal lattice (i-motif included in the connector strands for 1D or 2D) or shrink and expand (i-motif included in all connector strands).

## 6 Applications of DNA Origami Lattices

**Figure 4:**
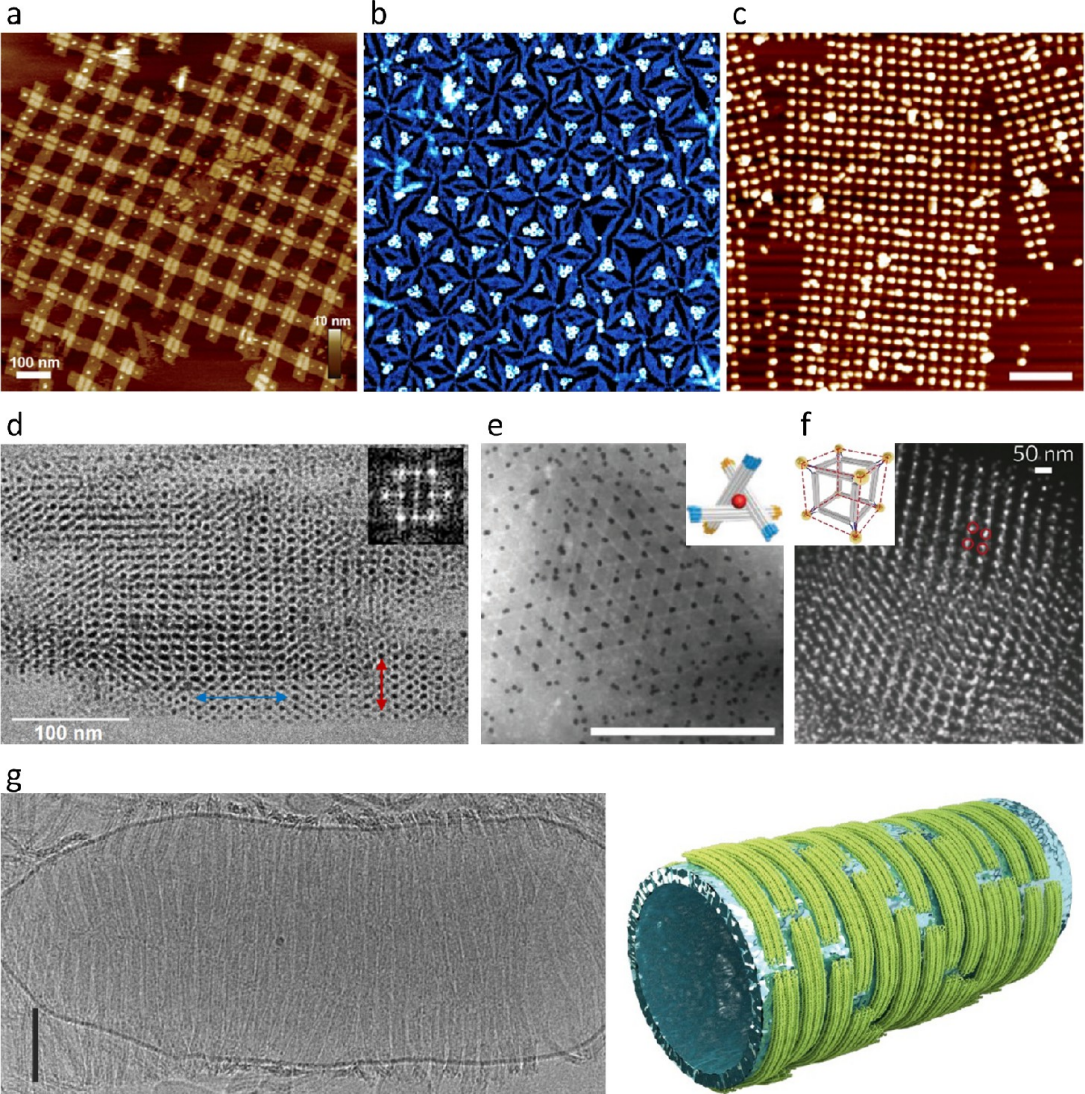
Applications of 2D and 3D DNA origami lattices. (a) AFM image of a lattice of cross-shaped DNA origami on mica decorated with streptavidin. (b) AFM image (1.1 × 1.1 μm^2^) of a lattice of DNA origami triangles on mica. The holes in the triangles have been filled with Redβ proteins via directed adsorption under conditions that resulted in about 70% of the holes featuring three ring-shaped Redβ complexes. (c) AFM image (scale bar 500 nm) of a lattice of cross-shaped DNA origami on mica decorated with AuNRs. (d) Cryo-TEM image of a tetragonal 3D AuNP lattice fabricated by electrostatic coassembly of 2.5 nm AuNPs and DNA origami six-helix bundles (6HBs). (e) TEM image (scale bar 500 nm) of a rhombohedral host–guest lattice assembled from DNA origami monomers with attached 20 nm AuNPs via shape-complementary blunt-end stacking. The inset shows a schematic representation of a single DNA origami monomer with the attached AuNP (red). (f) STEM image of a simple cubic hybrid lattice composed of DNA origami cubes assembled via connecting 10 nm AuNPs. The inset shows a schematic representation of a single DNA origami monomer with attached AuNPs (gold). (g) Cryo-TEM image (scale bar 100 nm) and schematic representation of a tubular lipid vesicle with a regular lattice of curved, beam-like DNA origami nanostructures at its surface. (a) Reprinted with permission from ref (42). Copyright 2014 John Wiley & Sons. (b) Reprinted with permission from ref (76). Copyright 2016 American Chemical Society. (c) Reprinted with permission from ref (78). Copyright 2021 American Chemical Society. (d) Reprinted with permission from ref (38). Published 2019 by The Royal Society of Chemistry. (e) Reprinted with permission from ref (33). Copyright 2018 John Wiley & Sons. (f) Reprinted with permission from ref (81). Copyright 2016 Springer Nature. (g) Reprinted with permission from ref (84). Published 2018 by Springer Nature.

The main application of DNA origami lattices so far is to use them as scaffolds for the arrangement of functional nanoscale entities. As already mentioned in Section 3, a rather straightforward approach was demonstrated by Aghebat Rafat et al., who decorated DNA origami cross shapes with biotin modifications, which were employed to specifically bind streptavidin after successful 2D lattice assembly at a mica surface (see Figure 4a).^42^ The same conceptual approach has also been used to fabricate quantum dot lattices.^46^ Similarly, also aptamer-modified DNA origami frames have been assembled into lattices on mica surfaces and filled with various proteins via multivalent binding.^75^

A different strategy for patterning proteins that also employs DNA origami lattices but does not rely on the availability of specific ligands was presented by Ramakrishnan et al.^76^ Here, 2D lattices of DNA origami triangles were assembled at mica surfaces and subsequently exposed to different protein solutions. The lattices acted as lithography masks that directed the nonspecific adsorption of the proteins to the exposed mica areas in the holes of the triangles (see Figure 4b). In this way, the authors fabricated regular patterns of different proteins, including Redβ, Redβ-GFP, Sak, ferritin, and bovine serum albumin (BSA). By adjusting the conditions of the protein adsorption step, they could also control the number of adsorbed proteins per hole in the mask and fabricate patterns of single proteins. Using the example of BSA, they furthermore demonstrated the Na^+^-induced desorption of the DNA origami mask after protein adsorption with the generated BSA pattern remaining intact. Since protein adsorption at the Mg^2+^-terminated mica surface is governed by electrostatic interactions, this approach can be extended to any negatively charged nanoscale object. This was subsequently demonstrated by Liu et al., who employed smaller DNA lattices composed of DNA tiles to direct the adsorption of negatively charged AuNPs.^77^ In an inverse approach, Yang et al. fabricated lattices of gold nanorods (AuNRs) by attaching DNA-coated AuNRs to DNA origami cross shapes.^78^ These AuNR-decorated DNA origami monomers were then assembled via blunt-end stacking into DNA origami lattices at mica surfaces (see Figure 4c). By controlling the orientation of the DNA origami monomers and their connections with neighboring ones through the tuning of the stacking contacts, different AuNR lattices could be achieved, including 1D arrays, isotropic 2D lattices, and anisotropic 2D lattices.

When it comes to applications of 3D DNA origami lattices, the current focus clearly lies on arranging AuNPs to generate photonic crystals. While such nanoparticle crystals can also be fabricated by the coassembly of AuNPs and DNA origami via electrostatic interactions (see Figure 4d),^38^ the majority of works employed DNA-coated AuNPs that were attached to the DNA nanostructures via hybridization.^79^ In this context, two different strategies can be distinguished. The first is conceptually similar to what has been described above for the 2D AuNR lattices assembled at the mica surface and involves the decoration of the DNA origami monomers with AuNPs. The decorated DNA origami monomers are then assembled into a 3D host–guest lattice (see Figure 4e).^33^ This strategy is rather versatile as it allows for the arrangement of nanoparticles encapsulated within DNA origami cages^80^ or chambers^62^ and can also be extended to other entities such as enzymes.^80^ In the alternative strategy, the DNA-coated nanoparticles themselves are integral parts of the assembling hybrid lattice and typically serve as connectors between the vertices of neighboring DNA origami monomers (see Figure 4f).^81^ Several different crystal lattices have been realized using this approach.^81^

So far, most of the explored applications of DNA origami lattices utilized static lattices. In this regard, understanding the assembly dynamics of the lattices is integral to controlling and optimizing the size, quality, and order of the fabricated 2D and 3D lattices. The few examples of applications so far that actually rely on the dynamics of lattice assembly were focused on controlling the shapes of lipid vesicles via the assembly of DNA origami lattices at their surfaces. Czogalla et al. first demonstrated that the assembly of planar yet bulky 3D DNA origami nanostructures at the surfaces of lipid vesicles may result in planar deformations of the vesicles.^82^ Here, vesicle attachment of the DNA origami was facilitated by cholesteryl modifications and sticky-end hybridization between two different DNA origami species via complementary staple overhangs was used to assemble the lattice. Other lattices based on the assembly of triskelion-like DNA origami monomers were not able to induce such macroscopic deformations of lipid vesicle shapes but only smaller, submicron deformations of lipid monolayers.^83^ Using curved beam-shaped DNA origami nanostructures displaying cholesteryl anchors on their concave surfaces, Franquelim et al. achieved drastic local alterations of lipid vesicle curvature.^84^ Furthermore, at high surface coverage, these DNA origami were found to assemble into periodic lattices and thereby transform spherical vesicles into ellipsoidal tubules (see Figure 4g). Notably, this lattice-induced formation of tubules occurred also in the absence of attractive interactions between the DNA origami such as sticky-end hybridization or blunt-end stacking.

## 7 Outlook

In summary, we have discussed the potential methods to assemble DNA origami-based building blocks into 2D and 3D lattices. The very fundamental features of such lattice assemblies and their dynamics have remained elusive, but currently, works that focus on understanding the processes of lattice assembly are coming increasingly into view. Along with the ever-expanding toolbox for DNA origami designs, the development of user-defined assemblies paves the way for high-quality lattices with macroscopic scales. For example, the automated design paradigms of various 2D DNA origami motifs presented by Bathe and co-workers may become extremely useful in such settings.^85−87^

We believe that these versatile platforms can be employed in various cost-effective 2D material fabrication schemes and applications. For example, DNA lattices can be used as tools for scalable nanomanufacturing; i.e., they can be used as masks in lithographic processing^88−90^ or as scaffolds for metallization and (bio)mineralization-based composite materials synthesis,^91−94^ thus enabling a variety of programmable inorganic assemblies.^7^ Moreover, the techniques discussed here allow precision patterning of DNA templates with, e.g., proteins,^95^ metal nanoparticles,^96^ and chromophores,^97^ as well as fabrication of optically intriguing substrates, such as polarimeters^98^ and metasurfaces.^99^ In addition to the static assemblies, foreseeable dynamic, reconfigurable stimuli-responsive lattices^100^ may find various uses, e.g., in sensing, diagnostics, and information relay.^101^

## References

[ref1] Bathe M.; Rothemund P. W. K. DNA Nanotechnology: A Foundation for Programmable Nanoscale Materials. MRS Bull. 2017, 42, 882–888. 10.1557/mrs.2017.279.

[ref2] Seeman N. C.; Sleiman H. F. DNA Nanotechnology. Nat. Rev. Mater. 2018, 3, 17068 10.1038/natrevmats.2017.68.

[ref3] Nummelin S.; Kommeri J.; Kostiainen M. A.; Linko V. Evolution of Structural DNA Nanotechnology. Adv. Mater. 2018, 30, 1703721 10.1002/adma.201703721. 29363798

[ref4] Hui L.; Bai R.; Liu H. DNA-Based Nanofabrication for Nanoelectronics. Adv. Funct. Mater. 2022, 32, 2112331 10.1002/adfm.202112331.

[ref5] Kuzyk A.; Jungmann R.; Acuna G. P.; Liu N. DNA Origami Route for Nanophotonics. ACS Photonics 2018, 5, 1151–1163. 10.1021/acsphotonics.7b01580. 30271812 PMC6156112

[ref6] Keller A.; Linko V. Challenges and Perspectives of DNA Nanostructures in Biomedicine. Angew. Chem., Int. Ed. 2020, 59, 15818–15833. 10.1002/anie.201916390. PMC7540699 32112664

[ref7] Heuer-Jungemann A.; Linko V. Engineering Inorganic Materials with DNA Nanostructures. ACS Cent. Sci. 2021, 7, 1969–1979. 10.1021/acscentsci.1c01272. 34963890 PMC8704036

[ref8] Rothemund P. W. K. Folding DNA to Create Nanoscale Shapes and Patterns. Nature 2006, 440, 297–302. 10.1038/nature04586. 16541064

[ref9] Dey S.; Fan C.; Gothelf K. V.; Li J.; Lin C.; Liu L.; Liu N.; Nijenhuis M. D. A.; Saccà B.; Simmel F. C.; et al. DNA Origami. Nat. Rev. Methods Primers 2021, 1, 13 10.1038/s43586-020-00009-8.

[ref10] Howorka S. DNA Nanoarchitectonics: Assembled DNA at Interfaces. Langmuir 2013, 29, 7344–7353. 10.1021/la3045785. 23373872

[ref11] Martynenko I. V.; Ruider V.; Dass M.; Liedl T.; Nickels P. C. DNA Origami Meets Bottom-Up Nanopatterning. ACS Nano 2021, 15, 10769–10774. 10.1021/acsnano.1c04297. 34255962 PMC8320526

[ref12] Heuer-Jungemann A.; Liedl T. From DNA Tiles to Functional DNA Materials. Trends Chem. 2019, 1, 799–814. 10.1016/j.trechm.2019.07.006.

[ref13] Chen Y.; Sun W.; Yang C.; Zhu Z. Scaling Up DNA Self-Assembly. ACS Appl. Bio Mater. 2020, 3, 2805–2815. 10.1021/acsabm.0c00035. 35025410

[ref14] Mao C.; Sun W.; Seeman N. C. Designed Two-Dimensional DNA Holliday Junction Arrays Visualized by Atomic Force Microscopy. J. Am. Chem. Soc. 1999, 121, 5437–5443. 10.1021/ja9900398.

[ref15] Winfree E.; Liu F.; Wenzler L. A.; Seeman N. C. Design and Self-Assembly of Two-Dimensional DNA Crystals. Nature 1998, 394, 539–544. 10.1038/28998. 9707114

[ref16] Zheng J.; Constantinou P. E.; Micheel C.; Alivisatos A. P.; Kiehl R. A.; Seeman N. C. Two-Dimensional Nanoparticle Arrays Show the Organizational Power of Robust DNA Motifs. Nano Lett. 2006, 6, 1502–1504. 10.1021/nl060994c. 16834438 PMC3465979

[ref17] Yan H.; Park S. H.; Finkelstein G.; Reif J. H.; LaBean T. H. DNA-Templated Self-Assembly of Protein Arrays and Highly Conductive Nanowires. Science 2003, 301, 1882–1884. 10.1126/science.1089389. 14512621

[ref18] Zhang J.; Liu Y.; Ke Y.; Yan H. Periodic Square-Like Gold Nanoparticle Arrays Templated by Self-Assembled 2D DNA Nanogrids on a Surface. Nano Lett. 2006, 6, 248–251. 10.1021/nl052210l. 16464044

[ref19] Hamada S.; Murata S. Substrate-Assisted Assembly of Interconnected Single-Duplex DNA Nanostructures. Angew. Chem., Int. Ed. 2009, 48, 6820–6823. 10.1002/anie.200902662. 19688799

[ref20] Sun X.; Ko S. H.; Zhang C.; Ribbe A. E.; Mao C. Surface-Mediated DNA Self-Assembly. J. Am. Chem. Soc. 2009, 131, 13248–13249. 10.1021/ja906475w. 19715316

[ref21] Jones M. R.; Seeman N. C.; Mirkin C. A. Programmable Materials and the Nature of the DNA Bond. Science 2015, 347, 1260901 10.1126/science.1260901. 25700524

[ref22] Linko V.; Kostiainen M. A. De Novo Nanomaterial Crystals from DNA Frameworks. Nat. Mater. 2020, 19, 706–707. 10.1038/s41563-020-0709-5. 32581348

[ref23] Benson E.; Mohammed A.; Gardell J.; Masich S.; Czeizler E.; Orponen P.; Högberg B. DNA Rendering of Polyhedral Meshes at the Nanoscale. Nature 2015, 523, 441–444. 10.1038/nature14586. 26201596

[ref24] Linko V.; Kostiainen M. A. Automated Design of DNA Origami. Nat. Biotechnol. 2016, 34, 826–827. 10.1038/nbt.3647. 27504776

[ref25] Piskunen P.; Nummelin S.; Shen B.; Kostiainen M. A.; Linko V. Increasing Complexity in Wireframe DNA Nanostructures. Molecules 2020, 25, 1823 10.3390/molecules25081823. 32316126 PMC7221932

[ref26] de Llano E.; Miao H.; Ahmadi Y.; Wilson A. J.; Beeby M.; Viola I.; Barisic I. Adenita: Interactive 3D Modelling and Visualization of DNA Nanostructures. Nucleic Acids Res. 2020, 48, 8269–8275. 10.1093/nar/gkaa593. 32692355 PMC7470936

[ref27] Jun H.; Wang X.; Parsons M. F.; Bricker W. P.; John T.; Li S.; Jackson S.; Chiu W.; Bathe M. Rapid Prototyping of Arbitrary 2D and 3D Wireframe DNA Origami. Nucleic Acids Res. 2021, 49, 10265–10274. 10.1093/nar/gkab762. 34508356 PMC8501967

[ref28] Glaser M.; Deb S.; Seier F.; Agrawal A.; Liedl T.; Douglas S.; Gupta M. K.; Smith D. M. The Art of Designing DNA Nanostructures with CAD Software. Molecules 2021, 26, 2287 10.3390/molecules26082287. 33920889 PMC8071251

[ref29] Parikka J. M.; Sokolowska K.; Markešević N.; Toppari J. J. Constructing Large 2D Lattices Out of DNA Tiles. Molecules 2021, 26, 1502 10.3390/molecules26061502. 33801952 PMC8000633

[ref30] Liu W.; Zhong H.; Wang R.; Seeman N. C. Crystalline Two-Dimensional DNA-Origami Arrays. Angew. Chem., Int. Ed. 2011, 50, 264–267. 10.1002/anie.201005911. PMC3463376 21053236

[ref31] Wang X.; Jun H.; Bathe M. Programming 2D Supramolecular Assemblies with Wireframe DNA Origami. J. Am. Chem. Soc. 2022, 144, 4403–4409. 10.1021/jacs.1c11332. 35230115

[ref32] Tikhomirov G.; Petersen P.; Qian L. Fractal Assembly of Micrometre-Scale DNA Origami Arrays with Arbitrary Patterns. Nature 2017, 552, 67–71. 10.1038/nature24655. 29219965

[ref33] Zhang T.; Hartl C.; Frank K.; Heuer-Jungemann A.; Fischer S.; Nickels P. C.; Nickel B.; Liedl T. 3D DNA Origami Crystals. Adv. Mater. 2018, 30, 1800273 10.1002/adma.201800273. 29774971

[ref34] Gerling T.; Wagenbauer K. F.; Neuner A. M.; Dietz H. Dynamic DNA Devices and Assemblies Formed by Shape Complementary, Non-Base Pairing 3D Components. Science 2015, 347, 1446–1452. 10.1126/science.aaa5372. 25814577

[ref35] Wagenbauer K. F.; Sigl C.; Dietz H. Gigadalton-Scale Shape-Programmable DNA Assemblies. Nature 2017, 552, 78–83. 10.1038/nature24651. 29219966

[ref36] Goetzfried M. A.; Vogele K.; Mückl A.; Kaiser M.; Holland N. B.; Simmel F. C.; Pirzer T. Periodic Operation of a Dynamic DNA Origami Structure Utilizing the Hydrophilic–Hydrophobic Phase-Transition of Stimulus-Sensitive Polypeptides. Small 2019, 15, 1903541 10.1002/smll.201903541. 31531953

[ref37] Jiang T.; Meyer T. A.; Modlin C.; Zuo X.; Conticello V. P.; Ke Y. Structurally Ordered Nanowire Formation from Co-Assembly of DNA Origami and Collagen-Mimetic Peptides. J. Am. Chem. Soc. 2017, 139, 14025–14028. 10.1021/jacs.7b08087. 28949522

[ref38] Julin S.; Korpi A.; Nonappa; Shen B.; Liljeström V.; Ikkala O.; Keller A.; Linko V.; Kostiainen M. A. DNA Origami Directed 3D Nanoparticle Superlattice *via* Electrostatic Assembly. Nanoscale 2019, 11, 4546–4551. 10.1039/C8NR09844A. 30806410

[ref39] Loescher S.; Walther A. Supracolloidal Self-Assembly of Divalent Janus 3D DNA Origami via Programmable Multivalent Host/Guest Interactions. Angew. Chem., Int. Ed. 2020, 59, 5515–5520. 10.1002/anie.201911795. PMC7154728 31814217

[ref40] Buchberger A.; Simmons C. R.; Fahmi N. E.; Freeman R.; Stephanopoulos N. Hierarchical Assembly of Nucleic Acid/Coiled-Coil Peptide Nanostructures. J. Am. Chem. Soc. 2020, 142, 1406–1416. 10.1021/jacs.9b11158. 31820959

[ref41] Woo S.; Rothemund P. W. K. Self-Assembly of Two-Dimensional DNA Origami Lattices Using Cation-Controlled Surface Diffusion. Nat. Commun. 2014, 5, 4889 10.1038/ncomms5889. 25205175

[ref42] Aghebat Rafat A.; Pirzer T.; Scheible M. B.; Kostina A.; Simmel F. C. Surface-Assisted Large-Scale Ordering of DNA Origami Tiles. Angew. Chem., Int. Ed. 2014, 53, 7665–7668. 10.1002/anie.201403965. 24894973

[ref43] Suzuki Y.; Endo M.; Sugiyama H. Lipid-Bilayer-Assisted Two-Dimensional Self-Assembly of DNA Origami Nanostructures. Nat. Commun. 2015, 6, 8052 10.1038/ncomms9052. 26310995 PMC4560778

[ref44] Yonamine Y.; Cervantes-Salguero K.; Minami K.; Kawamata I.; Nakanishi W.; Hill J. P.; Murata S.; Ariga K. Supramolecular 1-D Polymerization of DNA Origami through a Dynamic Process at the 2-Dimensionally Confined Air–Water Interface. Phys. Chem. Chem. Phys. 2016, 18, 12576–12581. 10.1039/C6CP01586G. 27091668

[ref45] Kielar C.; Ramakrishnan S.; Fricke S.; Grundmeier G.; Keller A. Dynamics of DNA Origami Lattice Formation at Solid–Liquid Interfaces. ACS Appl. Mater. Interfaces 2018, 10, 44844–44853. 10.1021/acsami.8b16047. 30501167

[ref46] Xin Y.; Shen B.; Kostiainen M. A.; Grundmeier G.; Castro M.; Linko V.; Keller A. Scaling Up DNA Origami Lattice Assembly. Chem.–Eur. J. 2021, 27, 8564–8571. 10.1002/chem.202100784. 33780583 PMC8252642

[ref47] Characterization of Amorphous and Crystalline Rough Surface: Principles and Applications, 1st ed.; Experimental Methods in the Physical Sciences, Vol. 37, Zhao Y.; Wang G.-C.; Lu T.-M., Eds.; Academic Press, 2020.

[ref48] Xin Y.; Ji X.; Grundmeier G.; Keller A. Dynamics of Lattice Defects in Mixed DNA Origami Monolayers. Nanoscale 2020, 12, 9733–9743. 10.1039/D0NR01252A. 32324191

[ref49] Xin Y.; Martinez Rivadeneira S.; Grundmeier G.; Castro M.; Keller A. Self-Assembly of Highly Ordered DNA Origami Lattices at Solid-Liquid Interfaces by Controlling Cation Binding and Exchange. Nano Res. 2020, 13, 3142–3150. 10.1007/s12274-020-2985-4.

[ref50] Gervois A.; Troadec J. P.; Lemaitre J. Universal Properties of Voronoi Tessellations of Hard Discs. J. Phys. A: Math. Gen. 1992, 25, 6169 10.1088/0305-4470/25/23/014.

[ref51] Lemaítre J.; Gervois A.; Troadec J. P.; Rivier N.; Ammi M.; Oger L.; Bideau D. Arrangement of Cells in Voronoi Tesselations of Monosize Packing of Discs. Philos. Mag. B 1993, 67, 347–362. 10.1080/13642819308220137.

[ref52] Aurenhammer F.; Klein R.; Lee D.-T. Voronoi Diagrams and Delaunay Triangulations, 1st ed.; World Scientific, 2013 10.1142/8685.

[ref53] Mingeot-Leclercq M.-P.; Deleu M.; Brasseur R.; Dufrêne Y. F. Atomic Force Microscopy of Supported Lipid Bilayers. Nat. Protoc. 2008, 3, 1654–1659. 10.1038/nprot.2008.149. 18833202

[ref54] Jackman J. A.; Cho N.-J. Supported Lipid Bilayer Formation: Beyond Vesicle Fusion. Langmuir 2020, 36, 1387–1400. 10.1021/acs.langmuir.9b03706. 31990559

[ref55] Endo M. Surface Assembly of DNA Origami on A Lipid Bilayer Observed Using High-Speed Atomic Force Microscopy. Molecules 2022, 27, 4224 10.3390/molecules27134224. 35807467 PMC9268156

[ref56] Langecker M.; Arnaut V.; List J.; Simmel F. C. DNA Nanostructures Interacting with Lipid Bilayer Membranes. Acc. Chem. Res. 2014, 47, 1807–1815. 10.1021/ar500051r. 24828105

[ref57] Suzuki Y.; Sugiyama H.; Endo M. Complexing DNA Origami Frameworks through Sequential Self-Assembly Based on Directed Docking. Angew. Chem., Int. Ed. 2018, 57, 7061–7065. 10.1002/anie.201801983. 29644771

[ref58] Sato Y.; Endo M.; Morita M.; Takinoue M.; Sugiyama H.; Murata S.; Nomura S. M.; Suzuki Y. Environment-Dependent Self-Assembly of DNA Origami Lattices on Phase-Separated Lipid Membranes. Adv. Mater. Interfaces 2018, 5, 1800437 10.1002/admi.201800437.

[ref59] Suzuki Y.; Kawamata I.; Watanabe K.; Mano E. Lipid Bilayer-Assisted Dynamic Self-Assembly of Hexagonal DNA Origami Blocks into Monolayer Crystalline Structures with Designed Geometries. iScience 2022, 25, 104292 10.1016/j.isci.2022.104292. 35573202 PMC9097702

[ref60] Kocabey S.; Kempter S.; List J.; Xing Y.; Bae W.; Schiffels D.; Shih W. M.; Simmel F. C.; Liedl T. Membrane-Assisted Growth of DNA Origami Nanostructure Arrays. ACS Nano 2015, 9, 3530–3539. 10.1021/acsnano.5b00161. 25734977 PMC4415451

[ref61] Kempter S.; Khmelinskaia A.; Strauss M. T.; Schwille P.; Jungmann R.; Liedl T.; Bae W. Single Particle Tracking and Super-Resolution Imaging of Membrane-Assisted Stop-and-Go Diffusion and Lattice Assembly of DNA Origami. ACS Nano 2019, 13, 996–1002. 10.1021/acsnano.8b04631. 30588792

[ref62] Lin Z.; Emamy H.; Minevich B.; Xiong Y.; Xiang S.; Kumar S.; Ke Y.; Gang O. Engineering Organization of DNA Nano-Chambers through Dimensionally Controlled and Multi-Sequence Encoded Differentiated Bonds. J. Am. Chem. Soc. 2020, 142, 17531–17542. 10.1021/jacs.0c07263. 32902966

[ref63] Yang S.; Liu W.; Wang R. Control of the Stepwise Assembly-Disassembly of DNA Origami Nanoclusters by pH Stimuli-Responsive DNA Triplexes. Nanoscale 2019, 11, 18026–18030. 10.1039/C9NR05047G. 31560004

[ref64] Wu N.; Willner I. pH-Stimulated Reconfiguration and Structural Isomerization of Origami Dimer and Trimer Systems. Nano Lett. 2016, 16, 6650–6655. 10.1021/acs.nanolett.6b03418. 27586163

[ref65] Yang Y.; Endo M.; Hidaka K.; Sugiyama H. Photo-Controllable DNA Origami Nanostructures Assembling into Predesigned Multiorientational Patterns. J. Am. Chem. Soc. 2012, 134, 20645–20653. 10.1021/ja307785r. 23210720

[ref66] Suzuki Y.; Endo M.; Yang Y.; Sugiyama H. Dynamic Assembly/Disassembly Processes of Photoresponsive DNA Origami Nanostructures Directly Visualized on a Lipid Membrane Surface. J. Am. Chem. Soc. 2014, 136, 1714–1717. 10.1021/ja4109819. 24428846

[ref67] Willner E. M.; Kamada Y.; Suzuki Y.; Emura T.; Hidaka K.; Dietz H.; Sugiyama H.; Endo M. Single-Molecule Observation of the Photoregulated Conformational Dynamics of DNA Origami Nanoscissors. Angew. Chem., Int. Ed. 2017, 56, 15324–15328. 10.1002/anie.201708722. 29044955

[ref68] Mishra S.; Park S.; Emura T.; Kumi H.; Sugiyama H.; Endo M. Photocontrolled DNA Origami Assembly by Using Two Photoswitches. Chem.–Eur. J. 2021, 27, 778–784. 10.1002/chem.202004135. 33063405

[ref69] Groeer S.; Walther A. Switchable Supracolloidal 3D DNA Origami Nanotubes Mediated through Fuel/Antifuel Reactions. Nanoscale 2020, 12, 16995–17004. 10.1039/D0NR04209A. 32780076 PMC7612458

[ref70] Groeer S.; Schumann K.; Loescher S.; Walther A. Molecular Communication Relays for Dynamic Cross-Regulation of Self-Sorting Fibrillar Self-Assemblies. Sci. Adv. 2021, 7, eabj5827 10.1126/sciadv.abj5827. 34818037 PMC8612681

[ref71] Ijäs H.; Nummelin S.; Shen B.; Kostiainen M. A.; Linko V. Dynamic DNA Origami Devices: from Strand-Displacement Reactions to External-Stimuli Responsive Systems. Int. J. Mol. Sci. 2018, 19, 2114 10.3390/ijms19072114. 30037005 PMC6073283

[ref72] Nummelin S.; Shen B.; Piskunen P.; Liu Q.; Kostiainen M. A.; Linko V. Robotic DNA Nanostructures. ACS Synth. Biol. 2020, 9, 1923–1940. 10.1021/acssynbio.0c00235. 32589832 PMC7467825

[ref73] Ma N.; Dai L.; Chen Z.; Ji M.; Wang Y.; Tian Y. Environment-Resistant DNA Origami Crystals Bridged by Rigid DNA Rods with Adjustable Unit Cells. Nano Lett. 2021, 21, 3581–3587. 10.1021/acs.nanolett.1c00607. 33821653

[ref74] Wang Y.; Yan X.; Zhou Z.; Ma N.; Tian Y. pH-Induced Symmetry Conversion of DNA Origami Lattices. Angew. Chem., Int. Ed. 2022, 61, e202208290 10.1002/anie.202208290. 35934673

[ref75] Aghebat Rafat A.; Sagredo S.; Thalhammer M.; Simmel F. C. Barcoded DNA Origami Structures for Multiplexed Optimization and Enrichment of DNA-Based Protein-Binding Cavities. Nat. Chem. 2020, 12, 852–859. 10.1038/s41557-020-0504-6. 32661410 PMC7116572

[ref76] Ramakrishnan S.; Subramaniam S.; Stewart A. F.; Grundmeier G.; Keller A. Regular Nanoscale Protein Patterns via Directed Adsorption through Self-Assembled DNA Origami Masks. ACS Appl. Mater. Interfaces 2016, 8, 31239–31247. 10.1021/acsami.6b10535. 27779405

[ref77] Liu L.; Zheng M.; Li Z.; Li Q.; Mao C. Patterning Nanoparticles with DNA Molds. ACS Appl. Mater. Interfaces 2019, 11, 13853–13858. 10.1021/acsami.8b22691. 30793605

[ref78] Yang S.; Liu W.; Zhang Y.; Wang R. Bottom-Up Fabrication of Large-Scale Gold Nanorod Arrays by Surface Diffusion-Mediated DNA Origami Assembly. ACS Appl. Mater. Interfaces 2021, 13, 50516–50523. 10.1021/acsami.1c13173. 34637259

[ref79] Julin S.; Nummelin S.; Kostiainen M. A.; Linko V. DNA Nanostructure-Directed Assembly of Metal Nanoparticle Superlattices. J. Nanopart. Res. 2018, 20, 119 10.1007/s11051-018-4225-3. 29950921 PMC5997120

[ref80] Tian Y.; Lhermitte J. R.; Bai L.; Vo T.; Xin H. L.; Li H.; Li R.; Fukuto M.; Yager K. G.; Kahn J. S.; et al. Ordered Three-Dimensional Nanomaterials Using DNA-Prescribed and Valence-Controlled Material Voxels. Nat. Mater. 2020, 19, 789–796. 10.1038/s41563-019-0550-x. 31932669

[ref81] Tian Y.; Zhang Y.; Wang T.; Xin H. L.; Li H.; Gang O. Lattice Engineering Through Nanoparticle–DNA Frameworks. Nat. Mater. 2016, 15, 654–661. 10.1038/nmat4571. 26901516 PMC5282967

[ref82] Czogalla A.; Kauert D. J.; Franquelim H. G.; Uzunova V.; Zhang Y.; Seidel R.; Schwille P. Amphipathic DNA Origami Nanoparticles to Scaffold and Deform Lipid Membrane Vesicles. Angew. Chem., Int. Ed. 2015, 54, 6501–6505. 10.1002/anie.201501173. 25882792

[ref83] Journot C. M. A.; Ramakrishna V.; Wallace M. I.; Turberfield A. J. Modifying Membrane Morphology and Interactions with DNA Origami Clathrin-Mimic Networks. ACS Nano 2019, 13, 9973–9979. 10.1021/acsnano.8b07734. 31418553 PMC6764109

[ref84] Franquelim H. G.; Khmelinskaia A.; Sobczak J.-P.; Dietz H.; Schwille P. Membrane Sculpting by Curved DNA Origami Scaffolds. Nat. Commun. 2018, 9, 811 10.1038/s41467-018-03198-9. 29476101 PMC5824810

[ref85] Jun H.; Zhang F.; Shepherd T.; Ratanalert S.; Qi X.; Yan H.; Bathe M. Autonomously Designed Free-Form 2D DNA Origami. Sci. Adv. 2019, 5, eaav0655 10.1126/sciadv.aav0655. 30613779 PMC6314877

[ref86] Jun H.; Wang X.; Bricker W. P.; Bathe M. Automated Sequence Design of 2D Wireframe DNA Origami with Honeycomb Edges. Nat. Commun. 2019, 10, 5419 10.1038/s41467-019-13457-y. 31780654 PMC6882874

[ref87] Wang X.; Li S.; Jun H.; John T.; Zhang K.; Fowler H.; Doye J. P.K.; Chiu W.; Bathe M. Planar 2D Wireframe DNA Origami. Sci. Adv. 2022, 8, eabn0039 10.1126/sciadv.abn0039. 35594345 PMC9122324

[ref88] Shen B.; Linko V.; Tapio K.; Pikker S.; Lemma T.; Gopinath A.; Gothelf K. V.; Kostiainen M. A.; Toppari J. J. Plasmonic Nanostructures Through DNA-Assisted Lithography. Sci. Adv. 2018, 4, eaap8978 10.1126/sciadv.aap8978. 29423446 PMC5804581

[ref89] Piskunen P.; Shen B.; Keller A.; Toppari J. J.; Kostiainen M. A.; Linko V. Biotemplated Lithography of Inorganic Nanostructures (BLIN) for Versatile Patterning of Functional Materials. ACS Appl. Nano Mater. 2021, 4, 529–538. 10.1021/acsanm.0c02849.

[ref90] Shen J.; Sun W.; Liu D.; Schaus T.; Yin P. Three-Dimensional Nanolithography Guided by DNA Modular Epitaxy. Nat. Mater. 2021, 20, 683–690. 10.1038/s41563-021-00930-7. 33846583

[ref91] Liu X.; Zhang F.; Jing X.; Pan M.; Liu P.; Li W.; Zhu B.; Li J.; Chen H.; Wang L.; et al. Complex Silica Composite Nanomaterials Templated with DNA Origami. Nature 2018, 559, 593–598. 10.1038/s41586-018-0332-7. 30013119

[ref92] Nguyen L.; Döblinger M.; Liedl T.; Heuer-Jungemann A. DNA-Origami-Templated Silica Growth by Sol–Gel Chemistry. Angew. Chem., Int. Ed. 2019, 58, 912–916. 10.1002/anie.201811323. 30398705

[ref93] Shani L.; Michelson A. N.; Minevich B.; Fleger Y.; Stern M.; Shaulov A.; Yeshurun Y.; Gang O. DNA-Assembled Superconducting 3D Nanoscale Architectures. Nat. Commun. 2020, 11, 5697 10.1038/s41467-020-19439-9. 33173061 PMC7656258

[ref94] Athanasiadou D.; Carneiro K. M. M. DNA Nanostructures as Templates for Biomineralization. Nat. Rev. Chem. 2021, 5, 93–108. 10.1038/s41570-020-00242-5. 37117611

[ref95] Zhang P.; Liu X.; Liu P.; Wang F.; Ariyama H.; Ando T.; Lin J.; Wang L.; Hu J.; Li B.; et al. Capturing Transient Antibody Conformations with DNA Origami Epitopes. Nat. Commun. 2020, 11, 3114 10.1038/s41467-020-16949-4. 32561744 PMC7305102

[ref96] Hartl C.; Frank K.; Amenitsch H.; Fischer S.; Liedl T.; Nickel B. Position Accuracy of Gold Nanoparticles on DNA Origami Structures Studied with Small-Angle X-ray Scattering. Nano Lett. 2018, 18, 2609–2615. 10.1021/acs.nanolett.8b00412. 29498287 PMC6544511

[ref97] Klein W. P.; Rolczynski B. S.; Oliver S. M.; Zadegan R.; Buckhout-White S.; Ancona M. G.; Cunningham P. D.; Melinger J. S.; Vora P. M.; Kuang W.; et al. DNA Origami Chromophore Scaffold Exploiting HomoFRET Energy Transport to Create Molecular Photonic Wires. ACS Appl. Nano Mater. 2020, 3, 3323–3336. 10.1021/acsanm.0c00038.

[ref98] Gopinath A.; Thachuk C.; Mitskovets A.; Atwater H. A.; Kirkpatrick D.; Rothemund P. W. K. Absolute and Arbitrary Orientation of Single-Molecule Shapes. Science 2021, 371, eabd6179 10.1126/science.abd6179. 33602826

[ref99] Arbabi A.; Horie Y.; Bagheri M.; Faraon A. Dielectric Metasurfaces for Complete Control of Phase and Polarization with Subwavelength Spatial Resolution and High Transmission. Nat. Nanotechnol. 2015, 10, 937–943. 10.1038/nnano.2015.186. 26322944

[ref100] Wang W.; Chen C.; Vecchioni S.; Zhang T.; Wu C.; Ohayon Y. P.; Sha R.; Seeman N. C.; Wei B. Reconfigurable Two-Dimensional DNA Lattices: Static and Dynamic Angle Control. Angew. Chem., Int. Ed. 2021, 60, 25781–25786. 10.1002/anie.202112487. PMC9649588 34596325

[ref101] Song J.; Li Z.; Wang P.; Meyer T.; Mao C.; Ke Y. Reconfiguration of DNA Molecular Arrays Driven by Information Relay. Science 2017, 357, eaan3377 10.1126/science.aan3377. 28642234

